# An artificial intelligence model of whole-slide pathology specimens differentiating cutaneous high-grade squamous proliferations

**DOI:** 10.1007/s00428-025-04272-6

**Published:** 2025-09-25

**Authors:** Anne Petzold, Anja Wessely, Michael Erdmann, Stefan Schliep, Stephan Schreml, Luis Carlos Rivera Monroy, Julio Vera, Konstantin Drexler, Dennis Niebel, Kinan Maurice Hayani, Franklin Kiesewetter, Carola Berking, Elias A. T. Koch, Markus V. Heppt

**Affiliations:** 1 Department of Dermatology, Deutsches Zentrum Immuntherapie (DZI), CCC Erlangen-EMN, Bavarian Cancer Research Center (BZKF), Uniklinikum Erlangen, Friedrich-Alexander-Universität Erlangen-Nürnberg (FAU), 91054 Erlangen, Germany; 2 Department of Dermatology, University Hospital Regensburg, Regensburg, Germany; 3 Pattern Recognition Lab, Friedrich-Alexander-Universität Erlangen-Nürnberg (FAU), 91058 Erlangen, Germany; 4 Department of Pathology, Wake Forest Baptist Health, Medical Center Boulevard, Winston-Salem, NC USA; 5 MVZ Pathology, Sozialstiftung Bamberg, 96015 Bamberg, Germany; 6 Dermpath München, Laboratory for Dermatopathology, Oral Pathology and Molecular Pathology, Munich, Germany

**Keywords:** Carcinoma, Squamous Cell, Warts, Pathology, Artificial Intelligence

## Abstract

Cutaneous squamous cell carcinoma (cSCC) and verruca vulgaris (VV) are skin conditions involving the proliferation of epidermal keratinocytes requiring fundamentally different treatments. Histological evaluation of highly differentiated squamous cell proliferations can be challenging, particularly in small or superficial samples. This study aims to improve diagnostic accuracy using an AI model to distinguish cSCC from VV. We developed a deep-learning model using clustering-constrained attention multiple instance learning (CLAM) to classify hematoxylin and eosin-stained whole-slide images (WSIs) as cSCC or VV. The dataset comprised 289 WSIs (n = 148 cSCC, n = 141 VV). Quality control was ensured through expert review: the training cohort was evaluated by four dermatopathologists, and the evaluation cohort by six additional experts. On the training set, the model achieved an AUROC of 0.99, with an accuracy of 94.9% for cSCC and 91.2% for VV. On the evaluation set, it reached an AUROC of 0.96, and accuracies of 82.4% (cSCC) and 97.4% (VV), similar to the average performance of individual dermatopathologists. We successfully trained and implemented an interpretable deep-learning-based weakly supervised model on WSIs distinguishing cSCC from VV, which could enhance AI-supported diagnostics in the future.

**Supplementary Information:**

The online version contains supplementary material available at 10.1007/s00428-025-04272-6.

## Introduction

Cutaneous squamous cell carcinoma (cSCC) is one of the most prevalent types of skin cancer, arising from malignant proliferation of epidermal keratinocytes [1]. Although the majority of cSCC cases are detected early and have a good prognosis, up to 10% of the tumors may progress to locally advanced or metastatic lesions [2]. Patients diagnosed with cSCC typically undergo surgical intervention as the primary treatment modality, with adjunctive radiotherapy only in the presence of high-risk features such as perineural invasion. In cases of locally advanced or metastatic lesions, systemic therapies may be employed [3].

In contrast, common warts or verruca vulgaris (VV) appear as hyperkeratotic papules with dark inclusions caused by human papillomavirus (HPV) infection of keratinocytes [4]. Transmission of the virus occurs through direct contact infection and can affect individuals of any age, with children being particularly susceptible. Children often do not require treatment because verruca vulgaris can be self-limiting. In other cases, VV is treated with salicylic acid, 5% imiquimod cream, cryotherapy, thermotherapy, or local injections [4].

Despite their clinical and prognostic differences, distinguishing between these entities solely based on histological examination can sometimes be challenging due to overlapping characteristics, particularly in small or superficial tissue samples [5]. Both entities may present with marked hyperparakeratosis and acanthosis, and in certain cases, keratinocytes within warts may appear enlarged and exhibit greater atypia, particularly under the influence of sun damage and HPV-related effects, especially when inflamed. Verrucous carcinoma, a rare variant of cSCC, is histologically characterized by narrow invaginations of the epidermis and a smooth, non-infiltrative base, which differentiates it from other subtypes of cSCC. However, its resemblance to VV, particularly in small or superficial shave biopsies, can make differentiation challenging, as both lesions share similar histologic features [6].

Since an inappropriate diagnosis may result in overtreatment or undertreatment, both having potentially serious long-term unnecessary consequences for affected patients, it is essential to precisely distinguish these lesions.

Recent advancements in artificial intelligence (AI) and machine learning have revolutionized medical diagnostics, including dermatopathology [7–10]. AI-driven algorithms, when trained on large datasets of histological images, demonstrate remarkable capabilities in recognizing subtle patterns and features that the human eye might overlook [11, 12]. Leveraging these technologies holds promise in enhancing the accuracy and efficiency of diagnosing cutaneous lesions and accelerating diagnostic workflow.

In this study, we aimed to support the distinction of cSCC versus VV on hematoxylin and eosin (H&E) stained whole-slide images (WSIs) with AI, and compared the performance of this model to a panel of six expert dermatopathologists.

## Results

### Dataset

**Fig. 1:**
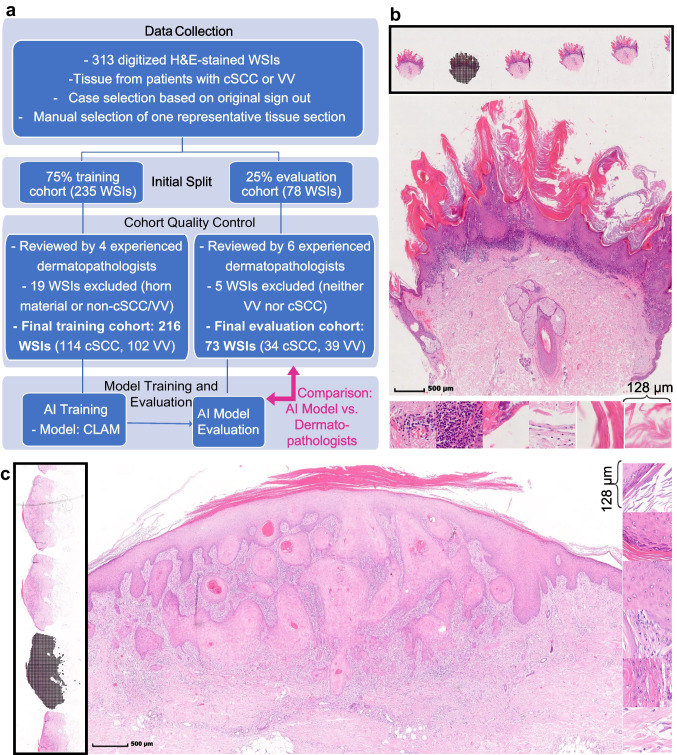
Data preprocessing workflow and tile extraction. **a** This flowchart illustrates the preprocessing steps applied to 313 H&E-stained whole slide images (WSIs) of tissue samples from patients with cutaneous squamous cell carcinoma (cSCC) or verrucae vulgaris (VV). After expert review, 216 WSIs (114 cSCC, 102 VV) were used for training and 73 WSIs (34 cSCC, 39 VV) for evaluation. A clustering constrained attention multiple instance learning (CLAM) model was trained and evaluated using these curated datasets. The model performance on the curated evaluation cohort was finally compared to the dermatopathologists´ diagnostic performance. **b-c** Representative WSIs of cSCC (**b**) and VV (**c**) demonstrate tile extraction. After selecting the best-quality tissue section using slideflow-studio, 256 × 256 px tiles (0.5 µm/pixel) were generated

A total of 313 digitized H&E-stained WSIs were initially collected, where a diagnosis of either cSCC or VV was made. Following review and curation of a consensus board of four expert dermatopathologists, 19 WSIs were excluded from the training cohort due to non-cSCC/VV diagnoses or the presence of only hyperkeratotic material. In four additional cases, the initial ground truth label was revised based on strong consensus among the consensus board. In the evaluation cohort, 5 WSIs were excluded due to non-target diagnoses. The final dataset thus consisted of 289 WSIs, comprising 148 cSCC samples from 135 patients and 141 VV samples from 132 patients. Of these, 216 WSIs (114 cSCC, 102 VV) were used for model training, and 73 WSIs (34 cSCC, 39 VV) formed the evaluation cohort (Fig. 1a). Representative examples of a cSCC and a VV case, including tile extraction for model input, are illustrated in Fig. 1b, c.

Patient and lesion characteristics are summarized in Table 1. Most cSCC samples originated from male patients (73.0%) with a mean age of 79.3 years (range: 51–94), and the majority of lesions were located on the head and neck (77.0%). Histologically, the tumors were predominantly well differentiated (G1: 80.4%). In contrast, VV samples showed a more balanced sex distribution (51.1% male) and a younger patient population with a mean age of 63.6 years (range: 5–95), reflecting the higher prevalence of VV in children. VV lesions were most commonly located on the head and neck (43.2%) or the extremities (37.6%).

**Table 1:** Baseline characteristics of the dataset. Abbreviations: cSCC: cutaneous squamous cell carcinoma, VV: verruca vulgaris, n.a.: not available, G1: grade 1, G2: grade 2

	cSCC (N = 148 samples of 135 patients)	VV (N = 141 samples of 132 patients)
Sex (male, female)	m: 73.0%; f: 27.0%	m: 51.1%; f: 48.9%
Mean age in years (range)	79.3 (51–94)	63.6 (5–95)
Localization	Head and neck: 114/148 = 77.0% Trunk: 10/148 = 6.8% Extremities: 22/148 = 14.9% Genital: 1/148 = 0.7% n.a.: 1/148 = 0.7%	Head and neck: 63/141 = 43.2% Trunk: 21/141 = 14.9% Extremities: 53/141 = 37.6% Genital: 2/141 = 1.4% n.a.: 2/141 = 1.4%
Grade of differentiation in cSCC (G1 or G2)	G1: 119/148 = 80.4% G2: 15/148 = 10.1% n.a.: 14/148 = 9.5%	n.a

### Training

**Fig. 2:**
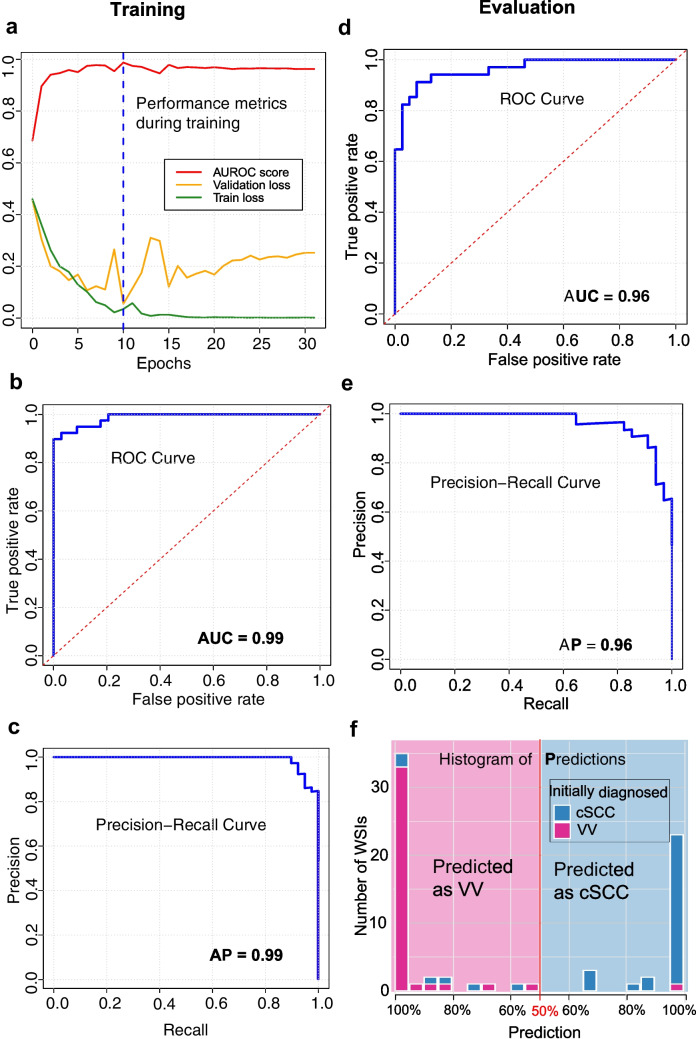
Model performance during training and evaluation. **a** The best model was identified at epoch 11 with a minimum validation loss of 0.153 (dotted line). AUROC = area under the receiver operating characteristics (ROC) curve. **b** (b) ROC curve and (**c**) precision-recall curve from epoch 11 in k-fold 1, showing classification performance between cutaneous squamous cell carcinoma (cSCC) and verruca vulgaris (VV). **d–e** ROC and precision-recall curves during final model evaluation on the test cohort. True/false positive rates and precision/recall reflect the model’s ability to distinguish cSCC from VV across thresholds. **f** Prediction histogram showing probability distributions for VV (left) and cSCC (right); most predictions were confidently assigned between 95–100%

The performance metrics during training in k-fold 1 are depicted in Fig. 2a: the best model was found in epoch 10 with a validation loss of 0.055. In this epoch, the training loss reached 0.035, while the area under the receiver operating characteristic curve (AUROC) score during validation was 0.99 (95% confidence interval (CI) 0.97–1), as illustrated by the corresponding ROC curve depicted in Fig. 2b. Additionally, the model achieved an average precision (AP) of 0.99 (95% CI 0.97–1), with the precision-recall curve visualized in Fig. 2c. The accuracy in predicting cSCC during validation was 94.9%, i.e., 37 out of 39 cSCC samples were identified correctly (95% CI 87.5–100%). For VV, 31 out of 34 samples were detected correctly, resulting in an accuracy of 91.2% (95% CI 80.6–100%).

For the training iterations in k-fold 2 and 3, comparable outcomes were observed. In k-fold 2, an accuracy of 86.8% (33 out of 38) was attained for cSCC, and 91.2% (31 out of 34) for VV. Similarly, in k-fold 3, accuracy rates of 89.5% (34 out of 38) for cSCC and 93.9% (31 out of 33) were achieved. In supplementary Figs. 1 and 2, the performance metrics during training, ROC, and precision-recall curves are depicted. However, the performance metrics of the model in k-fold 1 surpassed those of the other folds, making it the preferred choice. An example of correctly predicted tissue samples with respective attention heatmaps is depicted in supplementary Fig. 3 and 4 for cSCC and VV, respectively.

### Evaluation

**Fig. 3:**
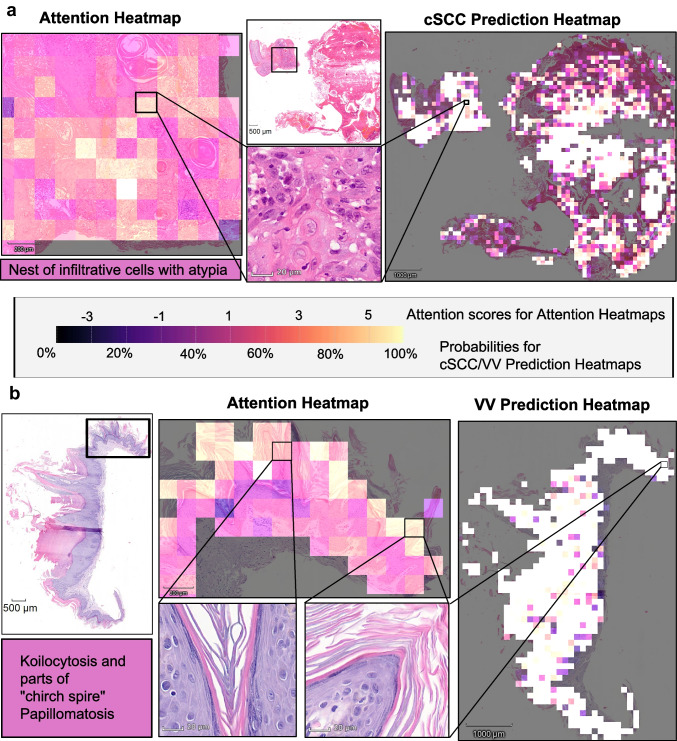
Model attention and class-specific prediction heatmaps for representative cases from the test set. Panels show interpretability outputs for a correctly predicted cSCC case (**a**, predicted probability: 82.4%) and a VV case (b, predicted probability: 99.9%). Attention heatmaps highlight regions most influential for the model's decision-making; prediction heatmaps visualize the spatial distribution of class probabilities (brighter = higher values). The central color scale applies to both. In (**a**), a high-attention tile—intentionally shown with partial overlap to better depict a nest of infiltrative atypical cells—illustrates a hallmark of cSCC. The cSCC prediction heatmap indicates higher probabilities within deeper dermal areas—corresponding to regions with invasive atypia—while keratinized epidermal zones appear darker, reflecting low cSCC probability. In (**b**), two exemplary high-attention tiles highlight church spire-like papillomatosis and koilocytosis, features typical for VV. The VV prediction heatmap shows a dense distribution of high-probability areas, consistent with the confident VV prediction (99.9%). These patterns suggest that the model attends to histologically relevant features

Evaluating the trained AI model with the final evaluation set, the model achieved an AUROC score of 0.96 (95% CI 0.92–1), and the average precision was 0.96 (95% CI 0.91–0.99) (Fig. 2d and e). The accuracy for predicting cSCC was 82.4% (95% CI 74.0–90.0%) (28 out of 34 cSCC samples were identified correctly) and 97.4% (95% CI 93.0—100%) for predicting VV (38 out of 39 VV samples were identified correctly). The distribution of prediction values for each WSI in the test dataset is depicted in the histogram in Fig. 2f: Most predictions ranged between 95 and 100% for both VV and cSCC. However, in two cases, the probability for cSCC was between 50 and 60%, indicating that these samples were ambiguous for the model. Examples of correctly predicted cSCC and VV tissue samples with respective attention and prediction heatmaps are depicted in Fig. 3. These heatmaps illustrate that the model focuses on histologically and diagnostically meaningful regions when making its decisions. In the shown cSCC case, areas with high attention and high predicted probability coincide with infiltrative nests of atypical keratinocytes, a key diagnostic feature of cSCC. In contrast, keratinized zones in the epidermis—although occasionally receiving high attention scores—showed low predicted probabilities for cSCC, reflecting their limited diagnostic specificity. In the VV example, attention is concentrated on regions displaying church spire papillomatosis and koilocytosis, which are characteristic features of VV. These patterns suggest that the model predominantly attends to diagnostically relevant structures, while still occasionally highlighting putatively less specific features.

On the external COBRA test set, the model correctly classified 43 of 50 cSCC cases, corresponding to an accuracy of 86%.

### Comparison of diagnostic performance to dermatopathologists

**Fig. 4:**
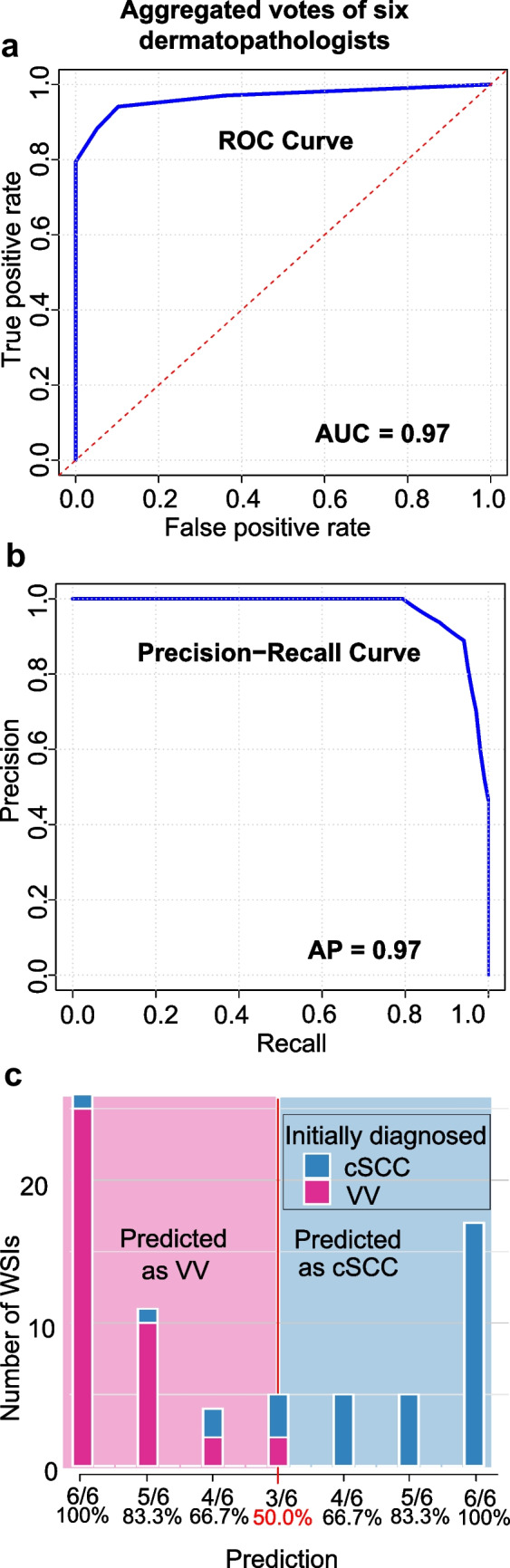
Consensus performance of six dermatopathologists on the evaluation cohort. **a** receiver operating characteristics (ROC) curve and (**b**) precision-recall curve based on aggregated votes across all 73 cases; both AUROC and average precision (AP) reached 0.97. **c** Histogram of consensus probabilities shows the distribution of predicted likelihoods for cutaneous squamous cell carcinoma (cSCC) and verruca vulgaris (VV)

To benchmark the AI model against expert performance, six dermatopathologists independently reviewed the entire evaluation cohort. The AUROC and AP of the consensus performance of the dermatopathologists were 0.97 (95% CI 0.92–1) and 0.97 (95% CI 0.93–1) (Fig. 4a and b). Full agreement among all six raters was achieved in only 43 out of 73 cases (58.9%), underscoring notable inter-rater variability. In the remaining 30 cases (41.1%), at least one dermatopathologist proposed a differing diagnosis, emphasizing the clinical complexity and relevance of this diagnostic task. Notably, in five cases (6.8%), the vote distribution was evenly split (3 vs. 3), resulting in a consensus probability of exactly 0.5 and no dominant classification (Fig. 4c). In four cases, the majority of the re-evaluating dermatopathologists favored a diagnosis of VV, whereas the original ground truth label was cSCC.

The AI model’s performance closely aligned with the consensus performance of the dermatopathologists (p = 0.841 for AUROC, and p = 0.828 for AP, see Supplementary Table 1). When compared to individual raters, the AI model showed a descriptively better performance than the average dermatologist (mean AP = 0.84, 95% CI 0.76–0.92). For class-specific accuracy, the model achieved 82.4% (95% CI 74.0–90.0%) for cSCC and 97.4% (95% CI 93.0–100%) for VV, compared to individual dermatopathologists with mean accuracies of 78.9% (95% CI 67.6–89.3%) for cSCC and 91.5% (95% CI 82.5–100%) for VV (Supplementary Table 1).

### Qualitative analysis of AI model misclassifications

A detailed review of the cases misclassified by the AI model revealed that most disagreements occurred in diagnostically challenging slides, some of which were also controversial among the human experts:

One slide that was originally labeled as VV but classified by the AI with 96.9% confidence as cSCC was also labeled as cSCC by 3 of the 6 dermatopathologists. Reviewers acknowledged that the diagnosis was difficult based on the available material.

In one case where the AI predicted VV (59.3% confidence) instead of the ground truth cSCC, one dermatopathologist agreed with the AI. The case was described as “initial cSCC or actinic keratosis with basal carcinomatous change; both options could be considered, depending on depth of invasion.”

Another case, initially labeled as cSCC, was predicted by the AI as VV with 99.7% confidence. All six dermatopathologists also labeled it as VV, suggesting a likely ground truth error.

In a case with an AI prediction of VV (99.9% confidence) despite a ground truth of cSCC, one dermatopathologist agreed with the AI. However, this case was likely a true misclassification by the model. Reviewers noted: “lesion tangentially sampled; step sections required.”

In three additional cases where the AI predicted VV instead of cSCC with confidence scores of 84.9%, 89.9%, and 74.5%, between two and three of the six dermatopathologists also favored VV, indicating borderline cases with uncertain ground truth.

Assuming the majority vote of the six evaluating dermatopathologists as the ground truth—and excluding the five cases with a 3:3 split—the AI model achieved an accuracy of 85.2% for cSCC and 95.1% for VV. Notably, all cases misclassified by the AI under this reference framework were also those with diagnostic ambiguity, as at least one dermatopathologist disagreed with the consensus. In contrast, all cases with unanimous agreement among the six dermatopathologists were correctly predicted by the AI model.

## Discussion

To date, histopathological diagnoses traditionally rely on visual assessment by dermatopathologists. In daily practice, distinguishing between closely related or similar conditions can be challenging. In such instances, immunohistochemical stains are commonly utilized as ancillary methods to distinguish between different diagnoses, such as PRAME for malignant melanoma [13]. However, immunohistochemistry, or even molecular pathology, are resource-intensive and may not always provide definitive diagnostic clarity. This is where morphology-driven AI can increasingly impact clinical practice and save resources including financial expenses, time, and human capital.

In this study, we developed a deep-learning model that demonstrated excellent performance during the evaluation, achieving an AUROC score of 0.96 (95% CI 0.92–1) and an accuracy of 82.4% (95% CI 74.0–90.0%) and 97.4% (95% CI 93.0–100%) for cSCC and VV, respectively. These results were clearly non-inferior to the mean diagnostic performance of individual dermatopathologists and closely matched the consensus performance of six experts. Furthermore, our model can generate attention heatmaps, thereby enhancing the interpretability and explainability of the AI decision, as well as assisting pathologists in ensuring focusing on crucial regions. Qualitative analysis of the heatmaps revealed that the model often attends to histologically meaningful structures—such as infiltrative atypical keratinocytes in cSCC, and koilocytosis or church spire-like papillomatosis in VV—suggesting alignment with known diagnostic features. However, we also observed that some keratinized areas, which represent a less specific finding, occasionally received high attention scores. This indicates that while the model captures relevant patterns, its focus is not exclusively limited to diagnostically decisive regions. The dataset was well balanced between cSCC and VV, and no manual annotations were necessary, allowing the model to generalize to entire slides without being trained solely on specific areas.

To ensure a robust ground truth, we incorporated several quality-control steps during data curation. The training cohort was independently reviewed by four dermatopathologists in a consensus board, and the evaluation cohort was assessed by six additional experts. This approach not only improved diagnostic reliability but also allowed us to quantify inter-observer variability. Notably, complete agreement among all six dermatopathologists was achieved in only 43 out of 73 evaluation cases, highlighting the real-world challenge and clinical relevance of distinguishing cSCC from VV, particularly in borderline cases. Interestingly, in four cases, the majority of the re-evaluating dermatopathologists favored a VV diagnosis, although the ground truth label was defined as cSCC. This may reflect a diagnostic bias in routine clinical practice, where pathologists may prefer diagnosing cSCC in equivocal cases to avoid underdiagnosing a malignant lesion. However, these ambiguities raise questions about the reliability of the original ground truth, especially in diagnostically difficult and borderline cases.

Our analysis of the AI model’s errors supports this notion. Several misclassified cases showed high internal prediction certainty (e.g., 99.7% probability for VV), and in some of these, all or most dermatopathologists also favored VV, despite the initial ground truth being defined as cSCC. When assuming the consensus vote of the re-evaluating dermatopathologists as the reference standard (excluding the five 3:3 cases), the model achieved an accuracy of 85.2% for cSCC and 95.1% for VV. All misclassified cases under this revised framework were again those with inter-pathologist disagreement, while all unanimously classified cases were correctly identified by the AI. These findings suggest that some of the so-called “errors” made by the model may actually reflect true inconsistencies or limitations in the human-assigned labels.

While clinical metadata such as age, sex, and lesion location were available and implicitly integrated into the original ground truth diagnoses from routine practice, the six dermatopathologists involved in the re-evaluation were deliberately blinded to this information. This design choice ensured a fair comparison with the AI model, which had access to histopathological images only. Although outcome data—such as local recurrence, metastasis, or overall patient survival— were not available for this study, we considered them of limited relevance for ground truth determination. Both entities typically have favorable clinical outcomes, and all cSCC cases included were highly differentiated (grade 1 or 2), which are associated with an excellent prognosis. While outcome-based validation could be valuable in principle, we found no indication in discordant cases that additional clinical information would have resolved the diagnostic uncertainty. This reinforces the importance of developing AI tools not as standalone diagnostic systems, but as decision-support systems, particularly valuable in ambiguous cases involving small or superficially sampled specimens. The model’s ability to provide an additional diagnostic perspective, especially when paired with expert review and potentially objective biological markers (e.g., genetic data), could help improve diagnostic reliability [14].

Comparing the results of this study to those of previously published AI classification studies in dermatopathology [15], the performance is similar. For instance, Del Amor et al. [16] proposed an attention-based weakly supervised method for diagnosing Spitzoid melanocytic lesions using only 51 WSIs and achieved a test accuracy of 80.0%. In another study by Xie et al. [17], 195 nevi and 117 melanomas were classified using various deep learning architectures and training methods, resulting in accuracies ranging from 96.7% to 99.9% and AUROC scores from 0.9944 to 0.9999. Jansen et al. [18] successfully used a modified UNet architecture to differentiate 14 different cutaneous adnexal tumors and distinguish them from more common cutaneous tumors. Despite very limited case numbers for training, their model achieved a total accuracy of 89.9% in the test set. A recent systematic review [19] summarized that the overall accuracy, sensitivity, and specificity of AI for tumor detection averaged 90%, 87%, and 91%, respectively, and criticized, among other aspects, the limited representation of cSCC.

Similarly, when comparing the diagnostic performance of deep learning models to that of dermatopathologists, our results align with previous studies in other dermatopathology fields, showing equal or superior performance of these models compared to the groups of pathologists involved in those studies [15].

Nevertheless, certain limitations must be acknowledged. The dataset was monocentric and digitized using a single slide scanner, which may affect the model’s generalizability to other institutions or imaging protocols. To partly address this, we performed an external evaluation on 50 well-differentiated cSCC cases from the publicly available COBRA dataset, achieving an accuracy of 86%. Although external verruca vulgaris cases were not available, this analysis suggests that the model retains diagnostic performance beyond the training cohort. Future validation on larger, multi-institutional datasets with diverse scanners and staining protocols will be essential to confirm robustness and general applicability [20]. Additionally, our model currently operates as a binary classifier, distinguishing only between VV and well to moderately differentiated cSCC. Other entities, such as actinic keratosis, keratoacanthoma, or poorly differentiated tumors, are not part of the training spectrum and could be misclassified. While this focused design was chosen deliberately as a proof-of-concept—targeting two morphologically similar but clinically distinct lesions—it limits the model’s immediate applicability in routine diagnostics. Expanding the model’s diagnostic breadth is therefore an important next step. A multi-class classification approach that includes additional clinically relevant mimickers could improve real-world performance. Such an expansion would require a substantially larger and more diverse dataset, ideally from multiple centers and encompassing a broader histological spectrum. Moreover, future work may benefit from strategies such as hierarchical classification, uncertainty estimation, or continual learning, which could allow the model to adapt incrementally to new diagnostic categories and reflect the complexity of dermatopathological practice.

## Materials & methods

### Dataset preparation

Digitized WSIs of tissue samples from patients with VV and well differentiated (grade 1) or moderately differentiated (grade 2) cSCC were collected from the dermatopathology unit of the University Hospital Erlangen, Germany, in 2023 (Fig. 1a). We only used H&E-stained tissue samples arising from different lesions. The cases represented a mixture of distinct biopsy techniques (spindle excision, punch biopsy, or curettage/shave biopsy), partial excisions, and complete excisions. In routine clinical practice, one or two board-certified dermatopathologists initially established the diagnosis of cSCC or VV, which served as the basis for the identification of eligible samples. All slides were scanned with the slide scanner Pannoramic 250 FLASH.

Since most of the WSIs contained multiple tissue sections, we manually selected one representative tissue section exhibiting the fewest artifacts. This selection process was performed by roughly circling the selected tissue section through the interactive tool *SlideFlow Studio *[21]. No additional annotations were applied.

As an initial data split, 75% of the data were randomly allocated for model training, while the remaining 25% were set aside as a held-out test set for performance evaluation. Within the training cohort, all selected WSIs were independently reviewed by four dermatopathologists to refine the diagnostic ground truth used for model development as a consensus diagnosis. Six dermatopathologists independently assessed the evaluation cohort. Their consensus served as a further reference standard for both quality control and to enable a final comparison with the AI model's performance (Fig. 1a).

In addition, to assess external generalizability, we included 50 histopathological images of well-differentiated (Grade 1/2) cSCC from the publicly available COBRA dataset (https://registry.opendata.aws/cobra/), selected by a board-certified dermatopathologist. These images were used as an independent test set.

As a next step, we extracted tiles from the images of size 256px x 256px, at 0.5 µm/pixel (128 µm). The tile extraction is illustrated in Fig. 1 with examples of a cSCC (Fig. 1b) and a VV (Fig. 1c). We used grayspace filtering as a tile-based method with a grayspace fraction of 0.6. Image tiles with a grayspace above this fraction were discarded.

### Network architecture, training and evaluation

We used clustering-constrained-attention multiple-instance learning (CLAM) [22] as a deep-learning-based weakly supervised method. CLAM automatically identifies subregions of high diagnostic value with attention-based learning and uses instance-level clustering to constrain and refine the feature space by learning from strongly and weakly attended tiles. Moreover, it has the capability to generate interpretable heatmaps for each slide to visualize the relative contribution and importance of every tissue region to the model's predictions without using any pixel-level annotations during training. CLAM is publicly available as an easy-to-use Python package over GitHub (https://github.com/mahmoodlab/CLAM).

To train and evaluate the CLAM model, image tiles had to be converted to features first. We therefore used the pathology-specific pretrained Phikon [23] feature extractor, a vision transformer pretrained on 40 million histology patches, that produced a 768-dimensional vector for each tile containing information about cellular structures, tissue morphology, staining patterns and many other specialized descriptive features. Bags of these feature vectors were generated for all slides in our dataset, serving as learning instances for the CLAM training.

In the CLAM training, an attention network ranks each region in a slide and assigns an attention score for each tile based on its relative importance to the slide-level diagnosis. These attention scores enable the generation of attention heatmaps. Additionally, strongly and weakly attended tiles are used as samples for instance-level clustering, that further supervise clustering layers to refine the feature space. In order to make the final prediction, the attention pooling function weights each tile by their respective attention score [22].

The training was executed using FastAI [24] with 1 cycle learning rate scheduling [25] which adjusts the learning rate during training in order to improve convergence and boosting overall model performance. Training was conducted over 32 epochs with an initial learning rate of 10^–4^, employing a threefold cross-validation approach. We selected these hyperparameters after testing various combinations, as they delivered the best performance in training and validation. During training, the cross-entropy loss of both training and validation as well as the AUROC score for validation was monitored.

The final model was evaluated on the held-out test dataset, generating probability scores between 0 and 1. A threshold of > 0.5 was used to classify cases as either cSCC or VV, and attention heatmaps were produced as part of the output. At the end of both training and evaluation, the model performance was described with AUROC, AP, and the accuracy of prediction for cSCC and VV. The project was carried out using the Python package *Slideflow* [21] on a NVIDIA GeForce RTX 4070 GPU.

### Comparison of the AI’s prediction performance to dermatopathologists

Six experienced dermatopathologists independently classified the evaluation cohort as cSCC or VV (binary). Their classification performance was assessed using the same metrics as those applied to the AI model—namely, AP, and class-specific accuracy. AUROC could not be meaningfully computed for individual raters, as their binary predictions do not allow for threshold-dependent analysis. For approximate comparability, AP was estimated as the average of precision and recall for each rater.

To enable a direct comparison with the AI model, we also computed performance metrics based on aggregated expert predictions. Specifically, the proportion of dermatopathologists who voted for cSCC was used as a continuous consensus score for each case (e.g., 4 out of 6 votes = 0.667), allowing the construction of ROC and precision-recall curves. In this context, the AUROC reflects the trade-off between sensitivity and specificity across varying consensus thresholds, and the AP captures the balance between precision and recall. Cases with a consensus score of exactly 0.5 (i.e., an equal split of votes) were excluded from class-specific accuracy calculations, as no majority decision could be established and the ground truth for these lesions ultimately remained unclear.

To estimate the uncertainty of performance metrics, 95% CIs for AUROC, AP, and class-specific accuracy were computed using nonparametric bootstrapping with 1,000 resamples. In each iteration, the evaluation set was sampled with replacement, and metrics were recalculated; the 2.5th and 97.5th percentiles defined the CI boundaries.

For formal significance testing, we compared AUROC between the AI model and the expert consensus using DeLong’s test. Differences in AP (AI vs. consensus and AI vs. individual raters) were evaluated using empirical p-values, defined as the proportion of bootstrap replicates in which the observed AP difference was at least as extreme as in the original data. Finally, differences in class-specific accuracy were assessed using McNemar’s test for paired categorical data. The analyses were conducted in R (version 4.3.0 with RStudio version 2022.07.2 + 576).

## Supplementary Information

Below is the link to the electronic supplementary material.

Supplementary file1 (PDF 114 KB)

Supplementary file2 (PDF 112 KB)

Supplementary file3 (PDF 380 KB)

Supplementary file4 (PDF 390 KB)

Supplementary file5 (DOCX 16 KB)

## Data Availability

The data that support the findings of this study are available from the corresponding author upon reasonable request.

## Abbreviations

cSCC: Cutaneous squamous cell carcinoma
VV: Verruca vulgaris
H&E: Hematoxylin and eosin
WSI: Whole slide image
AUROC: Area under the receiver operating characteristics curve
AP: Average precision
AI: Artificial intelligence
CLAM: Clustering constrained attention multiple instance learning
