# Supplementary figures and images for: An artificial intelligence model of whole-slide pathology specimens differentiating cutaneous high-grade squamous proliferations
Source: Virchows Arch. 2025 Sep 25;487(5):1047–58. doi: 10.1007/s00428-025-04272-6 (PMC12647221; doi:10.1007/s00428-025-04272-6)

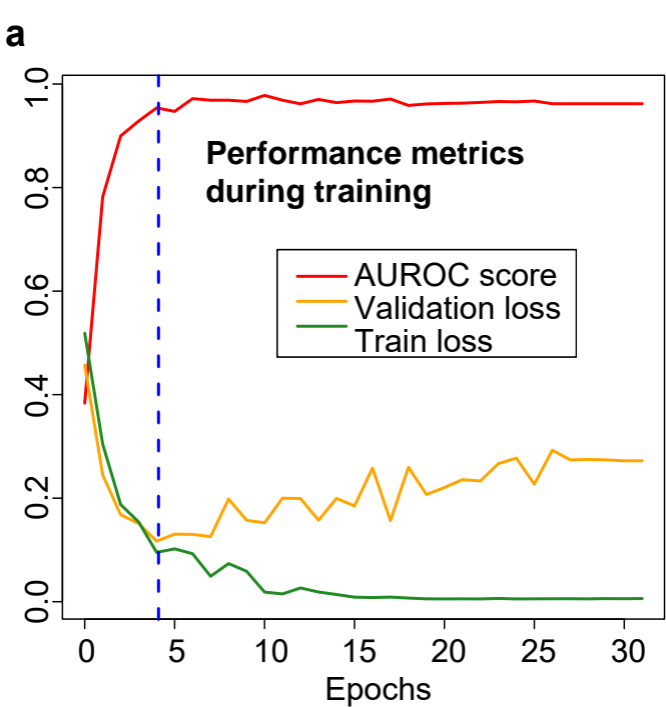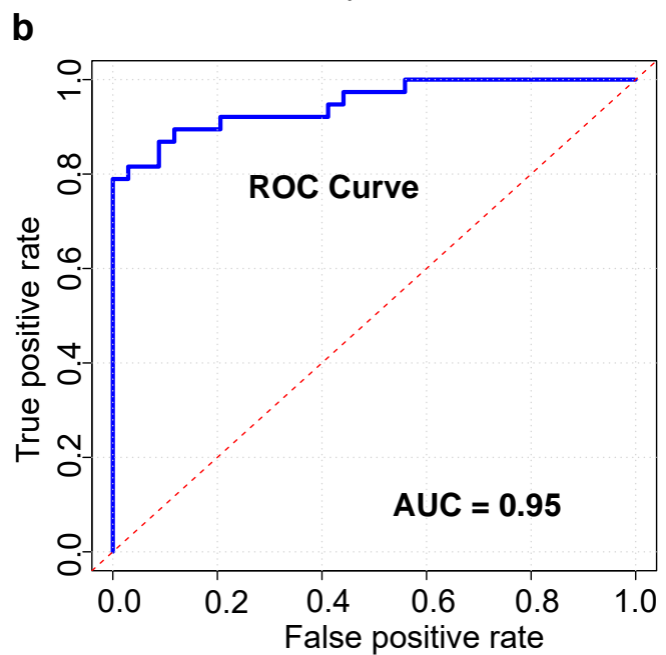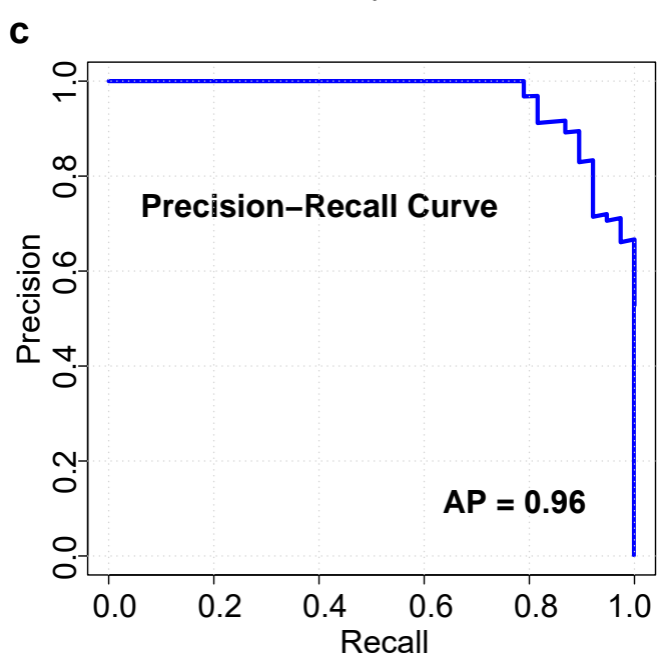

Supplement: Supplementary file 1 — Supplementary file1 (PDF 114 KB) [file 428_2025_4272_MOESM1_ESM.pdf]

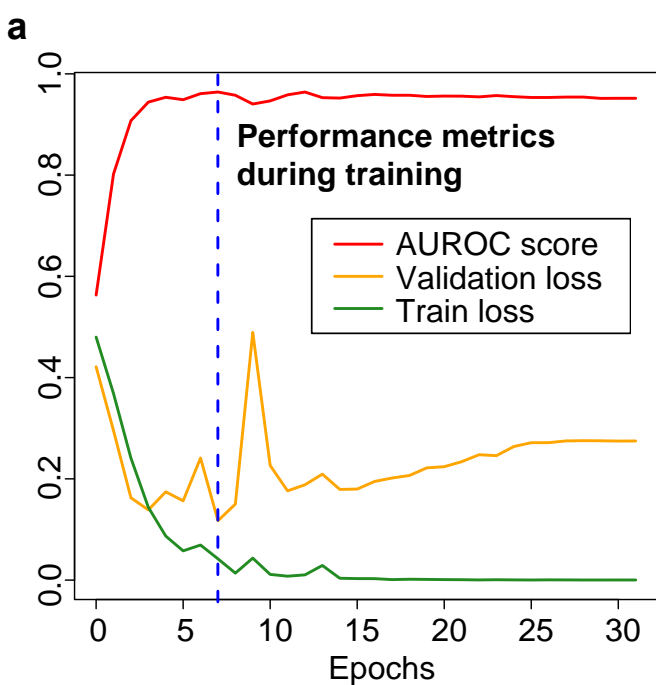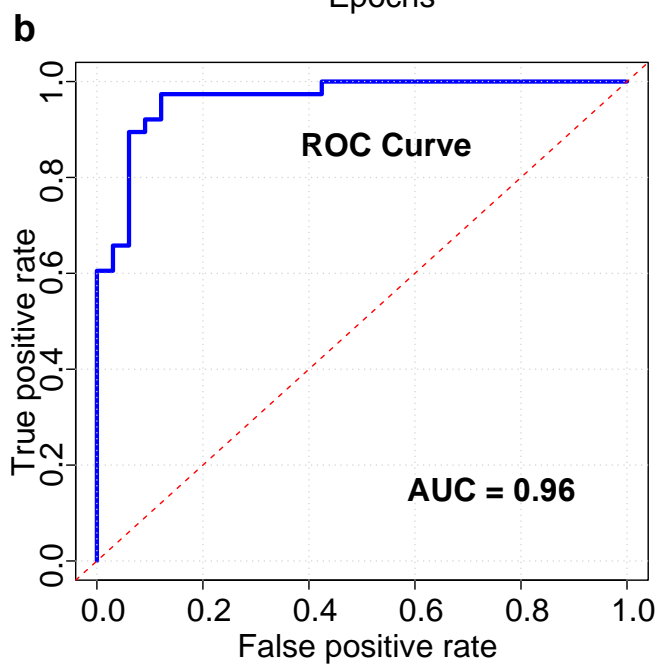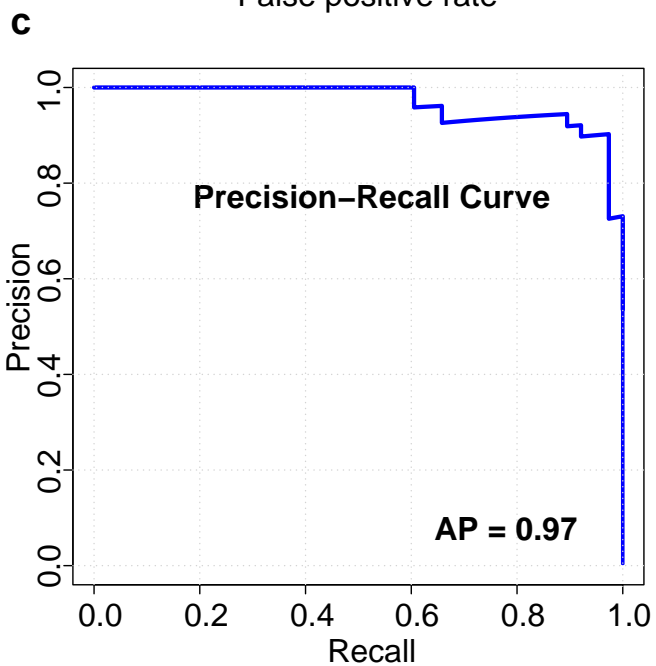

Supplement: Supplementary file 2 — Supplementary file2 (PDF 112 KB) [file 428_2025_4272_MOESM2_ESM.pdf]

**a**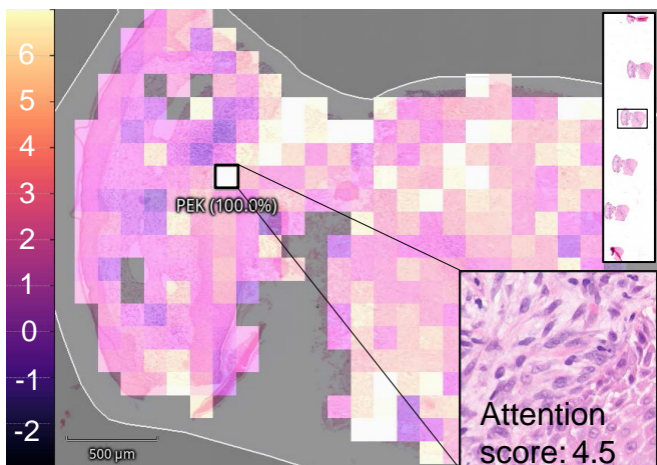**b**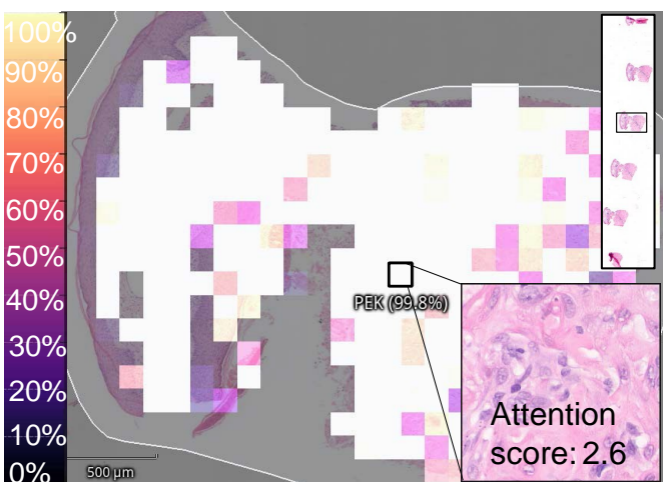**c**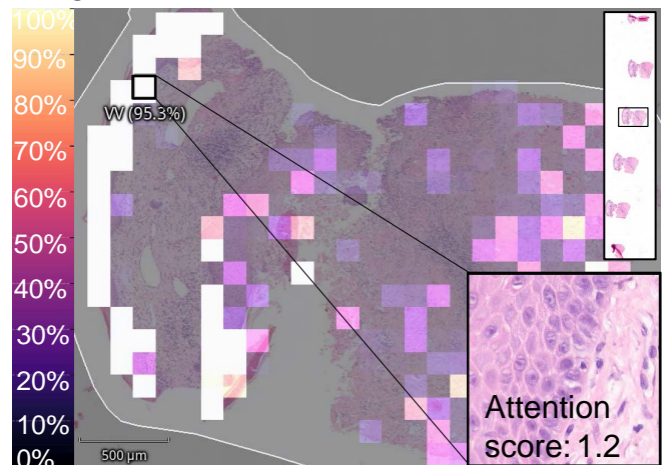

Supplement: Supplementary file 3 — Supplementary file3 (PDF 380 KB) [file 428_2025_4272_MOESM3_ESM.pdf]

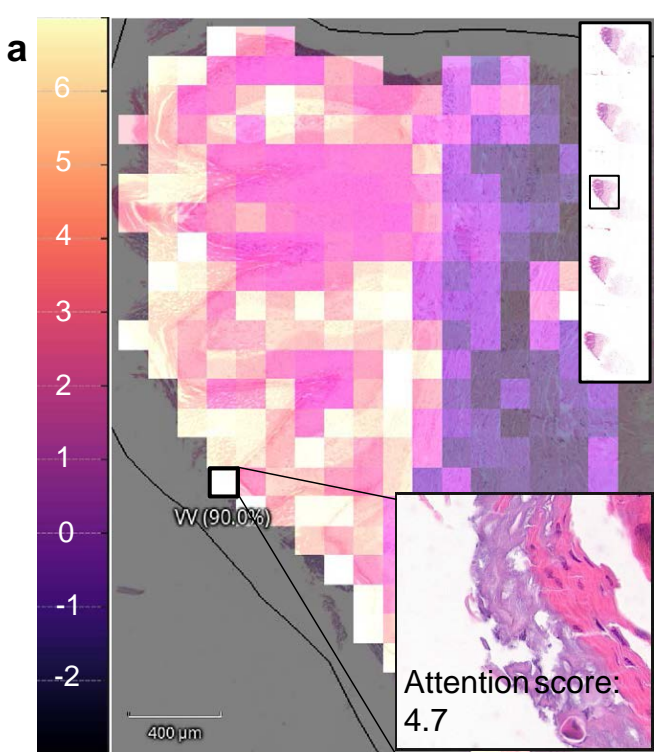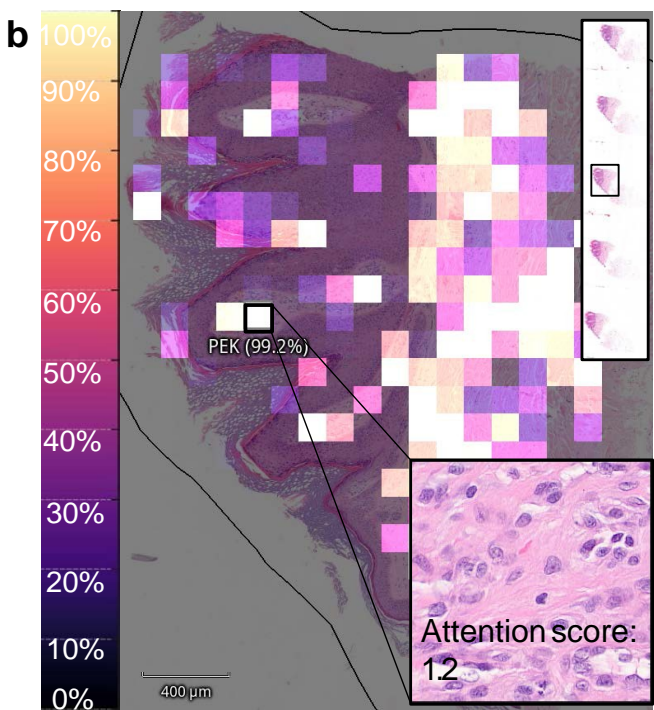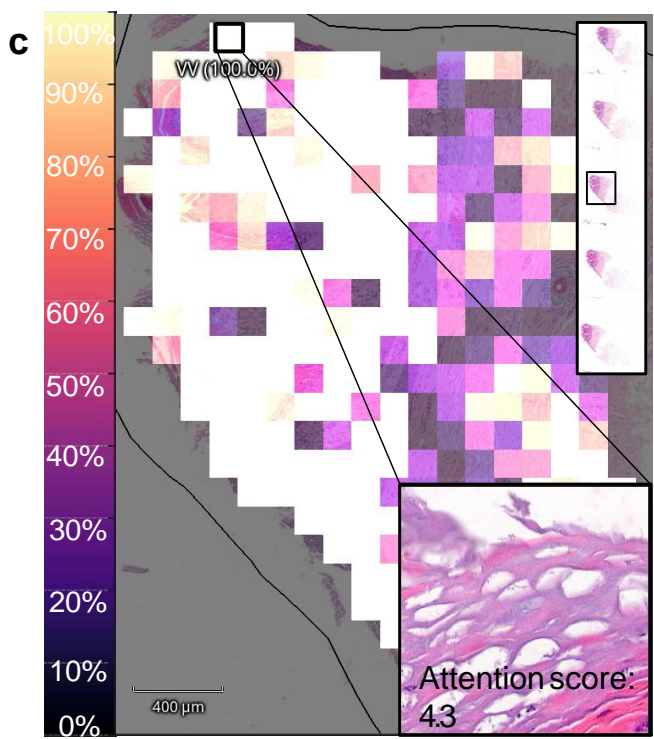

Supplement: Supplementary file 4 — Supplementary file4 (PDF 390 KB) [file 428_2025_4272_MOESM4_ESM.pdf]
