# Supplementary material for: An artificial intelligence model of whole-slide pathology specimens differentiating cutaneous high-grade squamous proliferations
Source: Virchows Arch. 2025 Sep 25;487(5):1047–58. doi: 10.1007/s00428-025-04272-6 (PMC12647221; doi:10.1007/s00428-025-04272-6)
Supplement: Supplementary file 5 — Supplementary file5 (DOCX 16 KB) [file 428_2025_4272_MOESM5_ESM.docx]

**Supplementary Table 1. Diagnostic performance of the AI model compared to dermatopathologists (with 95% confidence intervals)**

| **Model / Rater** | **AI model** | **Consensus** | **Rater 1** | **Rater 2** | **Rater 3** | **Rater 4** | **Rater 5** | **Rater 6** |
| --- | --- | --- | --- | --- | --- | --- | --- | --- |
| **AUROC (95% CI)** | 0.96 (0.92–1.00) | 0.97 (0.92–1.00) | — | — | — | — | — | — |
| **p-value** |  | 0.841* | — | — | — | — | — | — |
| **AP (95% CI)** | 0.96 (0.91–0.99) | 0.97 (0.93–1.00) | 0.82 (0.71–0.90) | 0.91 (0.83–0.97) | 0.86 (0.76–0.94) | 0.70 (0.56–0.83) | 0.90 (0.81–0.97) | 0.86 (0.77–0.94) |
| **p-value** |  | 0.828** | 0.462** | 0.475** | 0.489** | 0.49** | 0.478** | 0.469** |
| **Accuracy for cSCC (95% CI)** | 0.824 (0.74–0.90) | 0.861 (0.71-0.97) | 0.675 (0.52–0.84) | 0.911 (0.80–1.00) | 0.851 (0.73–0.96) | 0.678 (0.51–0.85) | 0.853 (0.72–0.97) | 0.757 (0.61–0.89) |
| **p-value** |  | NA*** | 0.131*** | 0.45*** | 1*** | 0.182*** | 1*** | 0.724*** |
| **Accuracy for VV (95% CI)** | 0.974 (0.93–1.00) | 0.947 (0.87-1.00) | 0.974 (0.91–1.00) | 0.923 (0.83–1.00) | 0.896 (0.80–0.98) | 0.772 (0.64–0.89) | 0.950 (0.87–1.00) | 0.975 (0.92–1.00) |
| **p-value** |  | 1*** | NA*** | 0.48*** | 0.371*** | 0.027*** | 1*** | 1*** |

* AUROC comparison and confidence intervals based on DeLong’s method.
** AP difference tested using non-parametric bootstrapping (1,000 resamples); empirical p-value reflects the proportion of replicates where the AP difference (AP_AI_ − AP_Consensus or Rater_) was at least as extreme as observed.
*** Class-specific accuracies compared using McNemar’s test; applied only when both models showed discordant predictions for the target class.

**Abbreviations:** AI = artificial intelligence, cSCC = cutaneous squamous cell carcinoma, VV = verruca vulgaris, CI = confidence interval, AUROC = area under the receiver operating characteristic curve, AP = average precision.
